# Initial presentation for acute low back pain: is early physical therapy associated with healthcare utilization and spending? A retrospective review of a National Database

**DOI:** 10.1186/s12913-022-08255-0

**Published:** 2022-07-02

**Authors:** Majd Marrache, Niyathi Prasad, Adam Margalit, Suresh K. Nayar, Matthew J. Best, Julie M. Fritz, Richard L. Skolasky

**Affiliations:** 1 Department of Orthopaedic Surgery, The Johns Hopkins University, 601 N. Caroline Street, JHOC 5223, Baltimore, MD 21287 USA; 2 Department of Physical Therapy & Athletic Training, University of Utah, Salt Lake City, UT USA

**Keywords:** Advanced imaging, Early physical therapy, Geographic differences, Healthcare utilization, Low back pain

## Abstract

**Background:**

Early initiation of physical therapy (PT) has been associated with lower healthcare costs and utilization; however, these studies have been limited to single institutions or healthcare systems. Our goal was to assess healthcare utilization and spending among patients who present for the first time with low back pain (LBP), according to whether they received early physical therapy (PT), using a large, nationwide sample; and geographic variation in rates of early PT and 30-day LBP-related spending.

**Methods:**

Using the Truven MarketScan database, we identified nearly 980,000 US adults ages 18–64 years who initially presented with acute LBP from 2010 through 2014 and did not have nonmusculoskeletal causes of LBP. Approximately 110,000 patients (11%) received early PT (≤2 weeks after presentation). We compared healthcare utilization and spending at 30 days and 1 year after presentation between patients who received early PT and those who did not. Alpha = 0.05.

**Results:**

At 30 days, early PT was associated with lower odds of chiropractor visits (odds ratio [OR] = 0.41, 95% confidence interval [CI] = 0.40–0.42), pain specialist visits (OR = 0.49, 95% CI = 0.47–0.51), emergency department visits (OR = 0.51, 95% CI = 0.49–0.54), advanced imaging (OR = 0.57, 95% CI = 0.56–0.58), orthopaedist visits (OR = 0.67, 95% CI = 0.66–0.69), and epidural steroid injections (OR = 0.68, 95% CI = 0.65–0.70). At 1 year, early PT was associated with less healthcare utilization. At 30 days, patients with early PT had lower mean LBP-related spending ($1180 ± $1500) compared with those without early PT ($1250 ± $2560) (*P* < 0.001). At 1 year, LBP-related spending was significantly less among patients who did not receive early PT ($2510 ± $3826) versus those who did ($2588 ± $3704). Early PT rates (range, 4–25%; *P* < 0.001) and 30-day LBP-related spending differed by state (range, $421 to −$410; *P* < 0.001).

**Conclusion:**

Early PT for acute LBP was associated with less 30-day and 1-year healthcare utilization and less 30-day LBP-related spending. Early PT rates and 30-day spending differed by US state.

**Level of evidence:**

IV

**Supplementary Information:**

The online version contains supplementary material available at 10.1186/s12913-022-08255-0.

## Background

Low back pain (LBP) affects a sizeable proportion of the US population, ranging from 1.4 to 20% [1], and accounts for substantial healthcare expenditures. The reported 1-year incidence of a first episode of LBP ranges from 6.3 to 15% [2]. The estimated cost of managing LBP in the US per year is between $12 billion and $91 billion, not accounting for indirect costs associated with loss of productivity and unemployment [3]. As the population ages, the prevalence of LBP is expected to increase, along with the associated costs of treating it [2]. Furthermore, with advances in imaging and treatments, the cost of managing LBP has increased substantially [4].

Current recommendations for initial treatment of acute LBP include physical therapy (PT) [5]. Earlier initiation of PT has been associated with less healthcare utilization and spending [6]; however, these studies have been limited to single institutions or health systems. Many patients with acute LBP receive late referrals to PT (i.e., at least 4 weeks after acute episode of injury) [7]. This delay could reflect certain practice guidelines that emphasize self-care and passive modalities as first-line treatment [8, 9]. Some physicians believe that most patients with LBP recover on their own within 2–4 weeks and that early PT could waste resources and risk “over-medicalizing” patients’ pain [10]. However, patients who do not receive PT may be more likely to seek care that may be costly and unnecessary [11].

Research is needed to evaluate the effects of early PT on healthcare utilization, particularly within the first 30 days after initial presentation for acute LBP, when they are most likely to experience pain and seek care. Our primary objectives were to compare 30-day and 1-year healthcare utilization and LBP-related spending among adults presenting for the first time with acute LBP according to whether they received early PT (within 2 weeks). Our secondary objectives were to assess geographic differences in early PT utilization and 30-day LBP-related spending. We hypothesized that early PT would be associated with less utilization of healthcare resources (i.e., advanced imaging, specialty appointments, emergency department [ED] visits, and epidural steroid injections [ESIs]) and lower LBP-related spending, and that we would find significant geographic variation in early PT utilization and 30-day spending.

## Methods

### Data source

We queried the Truven MarketScan commercial claims and encounters database to identify patients who presented to outpatient clinics for acute LBP from April 1, 2010, through December 31, 2014 [12]. The MarketScan database includes data for approximately 45 million US adult patients younger than 65 years with employer-based health insurance. The database contains inpatient claims, outpatient claims, and healthcare expenditures that are stored in a Health Insurance Portability and Accountability Act–compliant format. Our study was an analysis of secondary, deidentified data and was considered exempt from approval by our institutional review board.

### Patient selection

We included adults aged 18 to 64 years who presented for the first time during the study period with acute LBP [2, 13] (i.e., patients with no documented diagnosis of LBP within 3 months before the index visit). Patients were identified by using major diagnostic categories (diseases and disorders of the musculoskeletal system and connective tissue), provider type (internal medicine or family medicine), place of service (office or outpatient hospital), and *International Classification of Diseases, 9th Revision, Clinical Modification* (ICD-9-CM) codes for nonspecific LBP (SDC). We excluded patients with a concomitant diagnosis of a nonmusculoskeletal cause of LBP at the time of the index visit, including ankylosing spondylitis, cauda equina syndrome, cerebral palsy, endometriosis, gallstones, kidney stones, malignancy, osteomyelitis, paraplegia, Parkinson’s disease, pelvis fractures, pregnancy, quadriplegia, spinal cord injury, spine fracture, spine neoplasm, stroke, urinary tract infection, or uterine fibroids. We also excluded patients who did not have continuous health insurance enrollment at least 3 months prior to and 12 months after the index visit.

### Outcomes of interest

We assessed utilization of the following healthcare resources within 30 days and within 1 year after the index visit for LBP: advanced imaging, chiropractor visits, ED visits, epidural steroid injections (ESIs), orthopaedic surgeon clinic visits, and pain specialist clinic visits that were coded with major diagnostic category (MDC) equal to “08 – Disease and Disorders of the Musculoskeletal System and Connective Tissue.” These time points were selected to examine early vs. overall healthcare costs and utilization and to reflect our patient selection criterion of 12 months of continuous health insurance enrollment after the index visit. We determined healthcare utilization by using provider type and ICD-9-CM procedure codes.

We estimated LBP-related spending by calculating gross payments for all healthcare services, including PT, that were coded with MDC equal to “08 – Diseases and Disorders of the Musculoskeletal System and Connective Tissue” before applying coordination of benefits, deductibles, and copayments. Payments were adjusted to the 2014 US dollar by using the US Bureau of Labor Statistics Consumer Price Index [14].

Geographic differences in rates of early PT prescription (at least 1 PT session occurring within 2 weeks after the index visit for LBP) and 30-day LBP-related spending were also assessed by US state. We used the geographic regions defined by the US Census Bureau [15]: Northeast, South, Midwest, and West.

### Statistical analysis

We used Shapiro-Wilk tests to assess normality of continuous data. Continuous variables are described using means ± standard deviations, and categorical variables are described using numbers (percentages).

Multivariable regression adjusting for age, sex, and CCI value was performed to determine associations between early PT and healthcare costs and utilization. Generalized linear models with log link and gamma distribution for healthcare costs and Poisson regression models for healthcare utilization were used [16]. Odds ratios (ORs) and 95% confidence intervals (CIs) were calculated for each variable. After unadjusted analysis, probability of early PT utilization was estimated using logistic regression based on employment status (full-time, part-time, early retiree, or other), geographic location using employee 3-digit ZIP Code, and industry classification of the employer (mining/manufacturing, transportation/communications/utilities, or retail/finance/services). Estimates of healthcare costs and utilization were weighted by the inverse propensity score. To examine the effects of the weighting using the inverse propensity score, we compared the covariates before and after adjustment for propensity score.

Variation in regional early PT utilization was assessed by performing a chi-squared test. To assess geographic differences in spending, we subtracted the mean 30-day LBP-related spending among patients who received early PT from that among patients who did not. Mann-Whitney U tests were also used to assess geographic differences in 30-day spending between the 2 groups.

A sensitivity analysis was conducted that varied the required period of continuous health insurance enrollment prior to the index visit from 3 months to 6 months for the main comparison of total cost of care and healthcare utilization between those who did or did not receive early PT.

Significance was set at *P* < 0.05. All analyses were performed using SAS, version 9.4, software (SAS Institute, Cary, NC).

## Results

### Patient sample

We analyzed data of 979,223 patients (57% women; mean ± standard deviation age, 47 ± 11 years). Of these patients, 110,834 (11%) received early PT, with the first PT session occurring at a mean 5 ± 3 days after the index visit. Patients who received early PT were significantly more likely to be female and were slightly younger than those who did not receive early PT (Table 1). We calculated Charlson Comorbidity Index (CCI) values for each patient [17]. Among patients receiving early PT, a significantly smaller proportion had a CCI value ≥1 (1.8%) compared with those not receiving early PT (2.6%) (*P* < 0.001).

**Table 1:** Characteristics of 979,223 patients who presented for the first time with low back pain by receipt of early physical therapy, from the Truven MarketScan database (2010–2014)

Characteristics	N (%)		***P***
	Early PT, ***n*** = 110,834	No Early PT, ***n*** = 868,389	
Age, yr	46 ± 11^a^	47 ± 11^a^	< 0.001
Female sex	56,186 (51)	488,903 (56)	< 0.001
CCI value			
0	108,718 (98)	845,055 (97)	< 0.001
1	2055 (1.8)	22,488 (2.6)	
> 2	61 (0.06)	846 (0.1)	
Employment status			
Full-time	54,741 (49)	421,312 (48)	< 0.001
Part-time	1124 (1.0)	7679 (0.88)	
Early retiree	6259 (5.6)	47,675 (5.5)	
Other^b^	48,710 (44)	391,723 (45)	

*CCI* Charlson Comorbidity Index, *PT* physical therapy

^a^Reported as mean ± standard deviation

^b^Includes Medicare-eligible retiree, retiree, COBRA continuee, long-term disability recipient, surviving spouse, unknown

### 30-day healthcare utilization and LBP-related spending

On univariate analysis, the early PT group had a significantly lower incidence of advanced imaging, ESIs, chiropractor visits, orthopaedic surgeon and pain specialist clinic visits, and ED visits within 30 days compared with patients who did not receive early PT (all, *P* < 0.001; Table 2). After adjusting for age, sex, and CCI value, the early PT group had lower odds of advanced imaging (OR = 0.57, 95% CI = 0.56–0.58), chiropractor visits (OR = 0.41, 95% CI = 0.40–0.42), ED visits (OR = 0.51, 95%CI = 0.49–0.54), ESIs (OR = 0.68, 95%CI = 0.65–0.70), orthopaedic visits (OR = 0.67, 95% CI = 0.66–0.69), and pain specialist visits (OR = 0.49, 95% CI = 0.47–0.51). Significantly lower incidence of related healthcare use was observed in the models using inverse propensity score weighting (Table 2). Mean 30-day spending on LBP-related care was significantly lower among patients who received early PT ($747 ± $940) than among those who did not ($799 ± $1510) (*P* < 0.001). Similar differences were found in mean 30-day spending between those who received early PT and those who did not in inverse propensity score-weighted models.

**Table 2:** Thirty-day healthcare utilization among patients who presented for the first time with low back pain by receipt of early physical therapy, from the Truven MarketScan database (2010–2014)

Resource	N (%)		OR (95% CI)^**a**^	OR (95% CI)^**b**^
	Early PT, ***n*** = 110,834	No Early PT, ***n*** = 868,389		
Advanced imaging	14,572 (13)	182,971 (21)	0.57 (0.56–0.58)	0.59 (0.58–0.60)
Chiropractor visit	8469 (7.6)	146,073 (17)	0.41 (0.40–0.42)	0.43 (0.42–0.44)
ED visit	1775 (1.6)	26,772 (3.1)	0.51 (0.49–0.54)	0.54 (0.53–0.59)
ESI	3994 (3.6)	45,546 (5.2)	0.68 (0.65–0.70)	0.66 (0.62–0.70)
Orthopaedic visit	12,479 (11)	137,893 (16)	0.67 (0.66–0.69)	0.68 (0.67–0.70)
Pain specialist visit	3959 (3.6)	61,399 (7.1)	0.49 (0.47–0.51)	0.47 (0.43–0.50)
Total spending, $	747 ± 940^c^	799 ± 1510^c^		

*CI* confidence interval, *ED* emergency department, *ESI* epidural steroid injection, *OR* odds ratio, *PT* physical therapy

^a^Referent is patients who did not receive early PT

^b^Weighted by inverse propensity score based on employment status, geographic location, and industry classification of the employer

^c^Reported as mean ± standard deviation

### 1-year healthcare utilization and LBP-related spending

At 1 year after the index visit, patients in the early PT group had lower incidence of advanced imaging, ESIs, chiropractor visits, orthopaedic surgeon and pain specialist visits, and ED visits compared with patients who did not receive early PT (all, *P* < 0.001). After adjusting for age, sex, and CCI value, patients in the early PT group had lower odds of advanced imaging (OR = 0.77, 95% CI = 0.76–0.78), chiropractor visits (OR = 0.67, 95% CI = 0.65–0.68), ED visits (OR = 0.72, 95% CI = 0.71–0.73), ESIs (OR = 0.84, 95% CI = 0.83–0.86), orthopaedic visits (OR = 0.89, 95% CI = 0.87–0.90), and pain specialist visits (OR = 0.80, 95% CI = 0.79–0.81) (Table 3). Mean 1-year spending on LBP-related care was higher among patients who received early PT ($2588 ± $3704) than among those who did not ($2510 ± $3826) (*P* < 0.001). After inverse propensity score weighting, patients in the early PT group had lower incidence of related healthcare use and mean related healthcare costs.

**Table 3:** One-year healthcare utilization among patients who presented for the first time with low back pain by receipt of early physical therapy, from the Truven MarketScan database (2010–2014)

Resource	N (%)		OR (95% CI)^**a**^	OR (95% CI)^**b**^
	Early PT, ***n*** = 110,834	No Early PT, ***n*** = 868,389		
Advanced imaging	30,995 (28)	292,096 (34)	0.77 (0.76–0.78)	0.81 (0.80–0.82)
Chiropractor visit	19,802 (18)	213,803 (25)	0.67 (0.65–0.68)	0.65 (0.63–0.67)
ED visit	20,444 (18)	206,946 (24)	0.72 (0.71–0.73)	0.73 (0.72–0.75)
ESI	15,933 (14)	144,339 (17)	0.84 (0.83–0.86)	0.81 (0.78–0.84)
Orthopaedic visit	37,434 (34)	317,912 (37)	0.89 (0.87–0.90)	0.91 (0.88–0.92)
Pain specialist visit	26,331 (24)	243,812 (28)	0.80 (0.79–0.81)	0.74 (0.72–0.76)
Total spending, $	2588 ± 3704^c^	2510 ± 3826^c^		

*CI* confidence interval, *ED* emergency department, *ESI* epidural steroid injection, *OR* odds ratio, *PT* physical therapy

^a^Referent is patients who did not receive early PT

^b^Weighted by inverse propensity score based on employment status, geographic location, and industry classification of the employer

^c^Reported as mean ± standard deviation

### Sensitivity analysis

Adjusting the required period of continuous health insurance enrollment prior to the index visit to 6 months reduced the available sample size by 19% (*n* = 790,534 patients). Of these patients, 84,350 (11%) received PT within 14 days of the index visit.

Compared to those who did not receive early PT, these patients had lower 30-day spending on LBP-related care ($728 ± 814 vs. $791 ± 1701, *P* < 0.001) and reduced odds of receiving advanced imaging, chiropractor visits, ED visits, orthopaedic visits, and pain specialist visits (combined OR = 0.62, 95% CI = 0.61, 0.63, *P* < 0.001). Similarly, spending on LBP-related care ($2601 ± $4012 vs. $2714 ± 3917) and odds of use these other LBP-related healthcare (OR = 0.72, 95% CI = 0.71, 0.73) over 1 year were reduced in the group receiving early PT (*P* < 0.001).

### Geographic variations

**Fig. 1:**
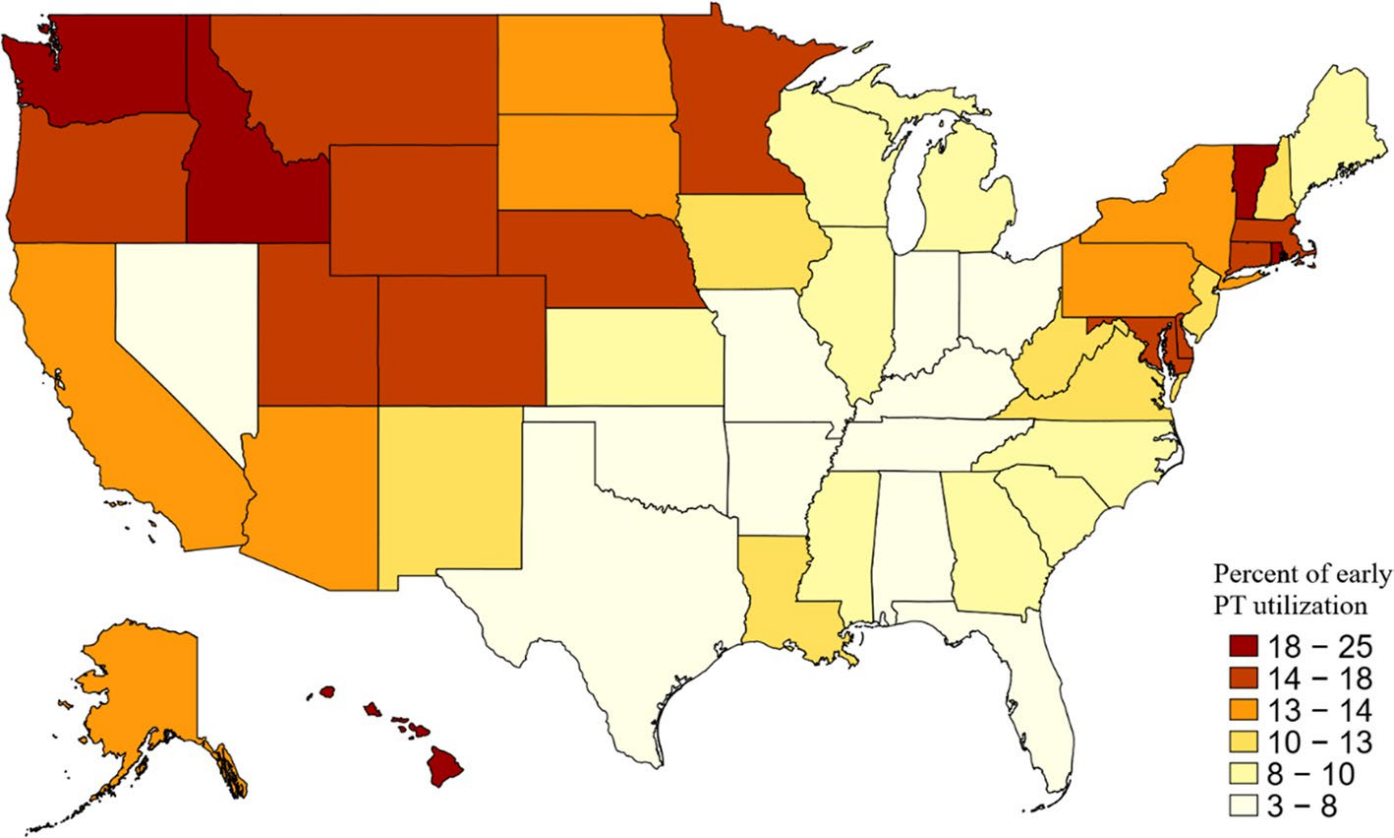
Early physical therapy utilization among 979,223 patients who presented for the first time with low back pain (LBP), by US state (*P* < 0.001). Early physical therapy (PT) was defined as at least 1 PT session occurring within 2 weeks after the index visit for LBP. Data were extracted from the Truven MarketScan database, 2010–2014. Map represents percentage of early PT utilization in patients who present for the first time with LBP, by state

Rates of early PT varied significantly among US states (*P* < 0.001). Florida had the lowest rate (4%), and Rhode Island had the highest (25%) (Fig. 1). The West had the highest rate of early PT prescription (16%), followed by the Northeast (15%), Midwest (9.4%), and South (8.6%) (*P* < 0.001).

**Fig. 2:**
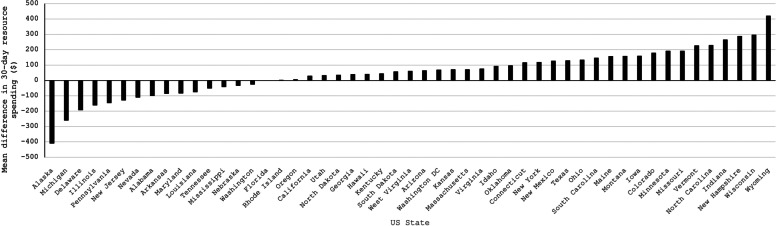
Mean differences in 30-day low back pain (LBP)–related spending among patients who presented for the first time with LBP who did not receive early physical therapy (PT) (*n* = 868,389) and those who did (*n* = 110,834), stratified by US state (*P* < 0.001). Early PT was defined as at least 1 PT session occurring within 2 weeks after the index visit for LBP. Data were extracted from the Truven MarketScan database, 2010–2014

Similarly, we found significant geographic differences in 30-day LBP-related spending between patients who did versus did not receive early PT (*P* < 0.01). Difference ranged from $421 in Wyoming to −$410 in Alaska (Fig. 2).

## Discussion

Our results are consistent with previous findings [11, 18, 19] that early PT in patients with acute LBP is associated with less healthcare utilization and spending. In our study of nearly 1 million patients, only 11% received PT within 2 weeks after their initial appointment. In our study of working-age adults (18–64 years of age), early PT was associated with less use of advanced imaging, ESIs, physician visits, and ED visits within 30 days and 1 year of the index visit compared with patients who did not receive early PT. LBP-related spending was significantly less among patients in the early PT group within 30 days after the index visit compared with those who did not receive early PT. However, at 1 year, LBP-related spending was higher among patients in the early PT group. We also found that rates of early PT utilization varied significantly by U.S. state and region.

We found a rate of early PT utilization of 11%, which is consistent with previous reports. Fritz et al. [19] reported that 7% of more than 32,000 patients with acute LBP received PT within 90 days of their index appointment; whereas, Childs et al. [18] found that 16% of more than 750,000 patients with acute LBP received PT within 90 days, with only 24% of those receiving PT initiating treatment within 2 weeks of their index appointment. The low rates of early PT in these studies may reflect the conflicting recommendations regarding treatment of acute LBP. The American Physical Therapy Association recommends PT during the acute episode of pain [9]; whereas, the American College of Physicians and American Pain Society recommend self-care and passive modalities as first-line treatment [8].

We found that the early PT group had lower odds of using advanced imaging during the 30 days and 1 year after the index visit. Previous studies reported similar results at up to 18 months (OR = 0.34) [19] and 2 years (OR = 0.52) [18] after the acute episode. Advanced imaging use is important because early use of advanced imaging has been associated with greater healthcare resource utilization. Webster and Cifuentes [20] determined that magnetic resonance imaging within 30 days after the onset of LBP was associated with greater risk of worse disability and higher medical costs and higher rates of surgery, regardless of the severity of pain. Therefore, for patients with LBP, early PT instead of imaging could decrease disability and spending.

Many subspecialties treat patients with LBP, but some specialties are more inclined to prescribe PT than others. Gellhorn et al. [7] found significant variation in PT referral rates between specialties, with physiatrists referring 32% of patients for PT, orthopaedic surgeons referring 22%, and generalists referring < 14%. They also found that patients who received early PT had fewer physician visits during the subsequent year (OR = 0.47) compared with those who did not receive early PT [7]. Fritz et al. [19] reached a similar conclusion with an OR for physician visits as low as 0.26 during the year after injury for patients with LBP who received early versus delayed PT. Our results are consistent with these findings, which show that early PT is associated with fewer specialist visits during the acute period of injury. This was also true during the year after the index visit.

LBP accounts for approximately 2.7 million visits to the ED in the US annually [21]. Whereas previous research showed no difference in ED utilization for patients who received early PT [3, 22], we found that early PT was associated with a lower odds of ED visits during the 30 days and 1 year after initial presentation. Pain outcomes for patients with LBP who seek care in the ED are generally poor, and patients are typically given a combination of nonsteroidal anti-inflammatory drugs, muscle relaxants, or opioid medications as a temporizing measure [23, 24].

For patients with acute LBP who have medically complex diagnoses or pain that has not responded to other nonoperative treatments, ESIs may be offered. We found an OR of 0.68 (95% CI = 0.65–0.70) for ESI during 30-day follow-up, which is likely low during the acute period of injury because ESIs are typically reserved for subacute (4–12 weeks) and chronic LBP. When extending follow-up beyond the acute phase, early PT is associated with a reduced need for an ESI for up to 2 years after initial presentation [7, 18, 19]. In a systematic review, the pooled OR for an ESI was 0.49 (95% CI = 0.41–0.58) when comparing early versus delayed PT referral from 1 to 2 years after initial presentation [11]. These results are similar to ours at 1 year after initial presentation, when we found that patients who had received early PT were still less likely to receive ESIs (OR = 0.84) than those who had not received early PT.

In terms of LBP-related spending, patients who received early PT spent significantly less at 30 days than patients who did not receive early PT. However, we found greater spending in the early PT group at 1 year compared with patients who did not receive early PT. This difference in 30-day and 1-year spending is likely because PT was included in the total cost analysis. Nevertheless, other studies have found significantly lower total LBP-related costs from 1 to 2 years after injury when early PT is used. Fritz et al. [19] found that patients who received early PT spent a mean $2700 less on LBP-related care than those who received delayed PT during the 18 months after injury. Childs et al. [18] found that mean total LBP-related costs for patients who received early PT were $1200 lower than those who received delayed PT at 2 years after their index appointment.

Rates of early PT varied significantly between states. More southern and midwestern states had early PT rates below the national average, and more northeastern states had early PT rates above the national average. These results are similar to those of Fritz et al. [19], who analyzed geographic variation of PT utilization within 90 days of onset of LBP, using the Midwest as the referent. They found that odds of PT utilization within 90 days after the onset of LBP were 1.6 times higher in the Northeast and West and 0.82 times lower in the South [19]. Other studies have shown geographic variation in the use of other LBP treatments, including imaging modalities, injections, opioids, and surgery [25–29]. Although provider density has been shown to be related to utilization of LBP services [4, 27], it is unclear why treatment patterns vary by region. Further research should examine the role that provider density, referral networks, and other health system factors may play in the availability of early PT for patients with LBP.

Strengths of our study include the use of a large database, which enabled us to include nearly 1 million patients—one of the largest cohorts analyzed for this topic. Furthermore, we extended our analysis to multiple specialties, revealing no difference in utilization of surgical and nonsurgical specialties during the 30 days after acute LBP. Also, setting our primary time period to 30 days reduced the likelihood that pain improved before treatment, allowing us to analyze healthcare utilization and spending during the acute phase. Our patient sample was restricted to those patients with an acute episode of LBP having no LBP-related encounters 3 months prior to the index visit (including physical therapy). Given the nature of employer-sponsored health insurance [30] (e.g., 42% of insured adults change their health insurance plan because of change in job or their employer changed plans), our sample size dropped precipitously if we extended this ‘look-back’ period to 12 months. When we extend our ‘look-back’ period to having no LBP-related encounters 12 months prior to the index visit, we experience a 33% reduction in sample size; however, the overall findings of the benefit of early PT remain. There may be difference in past healthcare experience between those who do or do not receive early PT (i.e., prior experience with physical therapy) that leads to improved clinical outcomes and, therefore, less overall utilization of healthcare services. We excluded patients with non-musculoskeletal causes of LBP, but some diagnoses and treatments may have been miscoded, leading to inaccuracies in the data that may affect our analysis. However, miscoding would be expected to occur at random and thus would have little effect on our results. We did not account for indirect or out-of-pocket costs for alternative care, such as acupuncture and massage, which has become more common for treating acute LBP. Therefore, we cannot determine whether early PT is associated with less use of these alternative, often costlier, services [31]. The database includes only patients with private insurance and not Medicare, and thus the results may not be generalizable to the entire U.S. population. Furthermore, we did not analyze associations between PT and prescription medications, which are often given as a temporizing measure during the acute phase of injury and could thus be affected by early referral to PT [23]. Finally, it is likely that patients who receive early PT are different in both known and unknown (unobservable) ways from those who do not receive early PT. These differences, particularly those unobservable differences, may impart selection bias in our study. In our statistical modeling of healthcare use and costs, we accounted for this potential bias through the use of inverse propensity score weighting. Propensity scores were based on characteristics that may be related to receipt of early PT but not related to the outcomes of interest. This does not replicate a randomized trial design but does attempt to mitigate the impact of selection bias on the inferences drawn. These limitations should be used to guide further research into the role that early PT plays in healthcare costs and utilization, such as the use of alternative care (e.g., acupuncture or massage) and the impact on the use of pain medications. Additionally, future research should focus on incorporating measures of patient health outcomes to determine not only the impact on cost and utilization but on patient health.

## Conclusion

These findings have important implications to guide healthcare policy when examining downstream healthcare costs and utilization with less use of advanced imaging, specialist visits, ED visits, and ESIs during the 30 days and 1 year after injury for patients receiving early PT compared with patients who did not receive PT during this early period. Furthermore, early PT was associated less LBP-related spending during the first 30 days after initial presentation, but higher spending during the year after the index visit. Rates of early PT utilization varied significantly by US state (ranging from 4 to 25%) and region (ranging from 9 to 16%).

## Supplementary Information

**Additional file 1.**

## Data Availability

Data from the Truven MarketScan database used for this study under a license agreement between Truven MarketScan and The Johns Hopkins University. The data that support the findings of this study are available from Truven MarketScan but restrictions apply to the availability of these data, which were used under license for the current study, and so are not publicly available. Data are, however, available from the authors upon reasonable request and with permission of Truven MarketScan.

## Abbreviations

CCI: Charlson Comorbidity Index
CI: Confidence interval
ED: Emergency department
ESI: Epidural steroid injection
ICD-9-CM: International Classification of Diseases, 9th Revision, Clinical Modification
LBP: Low back pain
OR: Odds ratio
PT: Physical therapy

## References

[1] Fatoye F, Gebrye T, Odeyemi I (2019). Real-world incidence and prevalence of low back pain using routinely collected data. Rheumatol Int.

[2] Hoy D, Brooks P, Blyth F, Buchbinder R (2010). The epidemiology of low back pain. Best Pract Res Clin Rheumatol.

[3] Dagenais S, Caro J, Haldeman S (2008). A systematic review of low back pain cost of illness studies in the United States and internationally. Spine J.

[4] Martin BI, Deyo RA, Mirza SK, Turner JA, Comstock BA, Hollingworth W (2008). Expenditures and health status among adults with back and neck problems. JAMA..

[5] Casazza BA (2012). Diagnosis and treatment of acute low back pain. AFP..

[6] Liu X, Hanney WJ, Masaracchio M, Kolber MJ, Zhao M, Spaulding AC (2018). Immediate physical therapy initiation in patients with acute low back pain is associated with a reduction in downstream health care utilization and costs. Phys Ther.

[7] Gellhorn AC, Chan L, Martin B, Friedly J (2012). Management patterns in acute low back pain: the role of physical therapy. Spine (Phila Pa 1976).

[8] Chou R, Qaseem A, Snow V, Casey D, Cross JT, Shekelle P (2007). Diagnosis and treatment of low back pain: a joint clinical practice guideline from the American College of Physicians and the American pain society. Ann Intern Med.

[9] Qaseem A, Wilt TJ, McLean RM, Forciea MA (2017). Noninvasive treatments for acute, subacute, and chronic low back pain: a clinical practice guideline from the American College of Physicians. Ann Intern Med.

[10] Von Korff M, Moore JC (2001). Stepped care for back pain: activating approaches for primary care. Ann Intern Med.

[11] Arnold E, La Barrie J, DaSilva L, Patti M, Goode A, Clewley D (2019). The effect of timing of physical therapy for acute low back pain on health services utilization: a systematic review. Arch Phys Med Rehabil.

[12] Truven Health Analytics MarketScan Research Databases. IBM/Watson Health - Truven Health Analytics. https://ictr.johnshopkins.edu/programs_resources/programs-resources/research-participant-recruitment-and-retention/truven-health-marketscan-research-databases/. Accessed 24 Dec 2019.

[13] Fritz JM, Magel JS, McFadden M, Asche C, Thackeray A, Meier W (2015). Early physical therapy vs usual care in patients with recent-onset low back pain: a randomized clinical trial. JAMA..

[14] U.S. Bureau of Labor Statistics: Archived Consumer Price Index Supplemental Files. U.S. Bureau of Labor Statistics. https://www.bls.gov/cpi/tables/supplemental-files/home.htm. Accessed 4 Mar 2020.

[15] United States Census Bureau: Census regions and divisions of the United States. United States Census Bureau https://www2.census.gov/geo/pdfs/maps-data/maps/reference/us_regdiv.pdf. Accessed 29 Apr 2020.

[16] Sroka CJ, Nagaraja HN (2018). Odds ratios from logistic, geometric, Poisson, and negative binomial regression models. BMC Med Res Methodol.

[17] Romano PS, Roos LL, Jollis JG (1993). Adapting a clinical comorbidity index for use with ICD-9-CM administrative data: differing perspectives. J Clin Epidemiol.

[18] Childs JD, Fritz JM, Wu SS, Flynn TW, Wainner RS, Robertson EK (2015). Implications of early and guideline adherent physical therapy for low back pain on utilization and costs. BMC Health Serv Res.

[19] Fritz JM, Childs JD, Wainner RS, Flynn TW. Primary care referral of patients with low back pain to physical therapy: impact on future health care utilization and costs. Spine (Phila Pa 1976). 2012;(37):2114–21. 10.1097/BRS.0b013e31825d32f5 22614792

[20] Webster BS, Cifuentes M (2010). Relationship of early magnetic resonance imaging for work-related acute low back pain with disability and medical utilization outcomes. J Occup Environ Med.

[21] Friedman BW, Chilstrom M, Bijur PE, Gallagher EJ (2010). Diagnostic testing and treatment of low back pain in US emergency departments. A national perspective. Spine (Phila Pa 1976).

[22] Karvelas DA, Rundell SD, Friedly JL, Gellhorn AC, Gold LS, Comstock BA (2017). Subsequent health-care utilization associated with early physical therapy for new episodes of low back pain in older adults. Spine J.

[23] Friedman BW, Dym AA, Davitt M, Holden L, Solorzano C, Esses D (2015). Naproxen with cyclobenzaprine, oxycodone/acetaminophen, or placebo for treating acute low back pain: a randomized clinical trial. JAMA..

[24] Friedman BW, O’Mahony S, Mulvey L, Davitt M, Choi H, Xia S (2012). One-week and 3-month outcomes after an emergency department visit for undifferentiated musculoskeletal low back pain. Ann Emerg Med.

[25] Cherkin DC, Deyo RA, Wheeler K, Ciol MA (1994). Physician variation in diagnostic testing for low back pain. Who you see is what you get. Arthritis Rheum.

[26] Deyo RA, Mirza SK (2006). Trends and variations in the use of spine surgery. Clin Orthop Relat Res.

[27] Friedly J, Chan L, Deyo R (2008). Geographic variation in epidural steroid injection use in medicare patients. J Bone Joint Surg Am.

[28] Webster BS, Cifuentes M, Verma S, Pransky G (2009). Geographic variation in opioid prescribing for acute, work-related, low back pain and associated factors: a multilevel analysis. Am J Ind Med.

[29] Weinstein JN, Lurie JD, Olson P, Bronner KK, Fisher ES, Morgan TS (2006). United States trends and regional variations in lumbar spine surgery: 1992–2003. Spine (Phila Pa 1976).

[30] Schoen C, Davis K. Listening to Workers: Findings from The Commonwealth Fund 1999 National Survey of workers’ health insurance. The Commonwealth Fund. https://www.commonwealthfund.org/publications/fund-reports/2000/jan/listening-workers-findings-commonwealth-fund-1999-national. Accessed 15 Dec 2021.

[31] Furlan AD, Yazdi F, Tsertsvadze A, Gross A, Van Tulder M, Santaguida L (2012). A systematic review and meta-analysis of efficacy, cost-effectiveness, and safety of selected complementary and alternative medicine for neck and low-back pain. Evid Based Complement Alternat Med.

