# Supplementary material for: Initial presentation for acute low back pain: is early physical therapy associated with healthcare utilization and spending? A retrospective review of a National Database
Source: BMC Health Serv Res. 2022 Jul 2;22:851. doi: 10.1186/s12913-022-08255-0 (PMC9250203; doi:10.1186/s12913-022-08255-0)
Supplement: Supplementary file 1 — Additional file 1. [file 12913_2022_8255_MOESM1_ESM.docx]

**SDC**

***International Classification of Diseases, 9th Revision, Clinical Modification* (ICD-9-CM) codes used as study inclusion and exclusion criteria**

| **Description** | **ICD-9-CM Codes** |
| --- | --- |
| *Inclusion Criteria* | |
| Low back pain | 71955, 7213, 7221, 72252, 72273, 72283, 72293, 72400, 72401, 72402, 72403, 72409, 7241, 7242, 7243, 7244, 7245, 7246, 72470, 72471, 72479, 7248, 7249, 7292, 7373, 75611, 75612, 8460, 8461, 8462, 8463, 8468,8469, 8472, 8473, 8479, 92231 |
| *Exclusion Criteria* | |
| Ankylosing spondylitis | 720.* |
| Cauda equina | 344.6* |
| Cerebral palsy | 343.9 |
| Urinary tract infection | 599.0 |
| Endometriosis | 218.* |
| Fracture spine/pelvis/femoral head | 805.*–809.*, 733.13–733.15, 733.96–733.98 |
| Gallstone | 574.* |
| Kidney stone | 592.* |
| Malignancy | 140.*–239.* |
| Osteomyelitis | 730.* |
| Paraplegia | 344.1 |
| Parkinson’s disease | 332 |
| Pregnant | V22.2 |
| Quadriplegia | 344 |
| Spinal cord injury | 952.* |
| Stroke | 434.91 |
| Uterine fibroids | 617.* |
